# Biotea: RDFizing PubMed Central in support for the paper as an interface to the Web of Data

**DOI:** 10.1186/2041-1480-4-S1-S5

**Published:** 2013-04-15

**Authors:** L Jael Garcia Castro, C McLaughlin, A Garcia

**Affiliations:** 1 Temporal Knowledge Bases Group, Department of Computer Languages and Systems, Universitat Jaumé I, Castello de la Plana, Valencia, 12071, Spain; 2 Institute for Digital Information and Scientific Communication, College of Communication and Information, Florida State University, Tallahassee, Florida, 32306-2651, USA

## Abstract

**Background:**

The World Wide Web has become a dissemination platform for scientific and non-scientific publications. However, most of the information remains locked up in discrete documents that are not always interconnected or machine-readable. The connectivity tissue provided by RDF technology has not yet been widely used to support the generation of self-describing, machine-readable documents.

**Results:**

In this paper, we present our approach to the generation of self-describing machine-readable scholarly documents. We understand the scientific document as an entry point and interface to the Web of Data. We have semantically processed the full-text, open-access subset of PubMed Central. Our RDF model and resulting dataset make extensive use of existing ontologies and semantic enrichment services. We expose our model, services, prototype, and datasets at http://biotea.idiginfo.org/

**Conclusions:**

The semantic processing of biomedical literature presented in this paper embeds documents within the Web of Data and facilitates the execution of concept-based queries against the entire digital library. Our approach delivers a flexible and adaptable set of tools for metadata enrichment and semantic processing of biomedical documents. Our model delivers a semantically rich and highly interconnected dataset with self-describing content so that software can make effective use of it.

## Background

For over 350 years, scientific publications have been fundamental to advancing science. Since the first scholarly journals, Philosophical Transactions of the Royal Society (of London) and the Journal de Sçavans, scientific papers have been the primary, formal means by which scholars have communicated their work, e.g., hypotheses, methods, results, experiments, etc. [1]. Advances in technology have made it possible for the scientific article to adopt electronic dissemination channels, from paper-based journals to purely electronic formats. By the same token, scholarly communication has been complemented by the adoption of blogs, mailing lists, social networks, and other technologies that in combination support the tissue, by means of which scholars communicate their work and establish connections with one another. However, in spite of the advances, scientific publications remain poorly connected to each other as well as to external resources. Furthermore, most of the information remains locked up in discrete documents without machine-processable content. Such interconnectedness and structuring would facilitate interoperability across documents as well as between publications and online resources resources available online. Scholarly data and documents are of most value when they are interconnected rather than independent [2].

In an effort to add value to the content of scientific publications, publishers are actively improving programmatic access to their products. For instance, Nature Publishing Group (NPG) recently released 20 million Resource Description Framework (RDF) statements, including primary metadata for more than 450,000 articles published by NPG since 1869. In this first release, the dataset includes basic citation information (title, author, publication date, etc.), identifiers, and Medical Subject Headings (MeSH) terms. Their data model makes use of vocabularies such as the Bibliographic Ontology (BIBO) [3], Dublin Core Metadata Initiative (DCMI) [4,5], Friend of a Friend (FOAF) [6,7], and the Publishing Requirements for Industry Standard Metadata (PRISM) [8] as well as ontologies that are specific to NPG [9]. Similarly, Elsevier provides an Application Programming Interface (API) that makes it possible for developers to build specialized applications [10].

Semantic Digital Libraries (SDLs) aim at applying semantic technologies in order to provide uniform access to metadata as well as machine-processable content; in such a way, SDLs intend to better support information retrieval and classification tasks [11,12]. Within the context of SDLs, ontologies can be used to: (i) organize bibliographic descriptions, (ii) represent and expose document contents, and (iii) share knowledge amongst users [12]. Recent efforts such as JeromeDL [11] allow users to semantically annotate books, papers, and resources. Similarly, the Bricks project [13] aims to integrate existing digital resources into a shared digital memory. It relies on OWL-DL in order to support, organize, and manage metadata. Efforts such as DOMEO [14] and the Living Document [15] illustrate how Semantic and Social Web technologies are being used in digital libraries within the biomedical domain. DOMEO is a web component developed using the Google Web Toolkit (GWT) and JavaScript. It allows users to manually or semi-automatically create unstructured or semi-structured semantic annotations that can be private, shared within selected groups, or made public. The Living Document (LD) makes use of the document as an interface to the Web of Data (WoD), a self-descriptive document fully interoperable with the Web. The LD also acts as a document router, operating by means of structured and organized social tagging and using existing ontologies. A desktop tool rather than a digital library, UTOPIA [16] is a Portable Document Format (PDF) reader that combines Semantic and Social Web principles with visualization tools and online content. Similar to DOMEO and LD, UTOPIA also aims to improve interoperability and user experience.

In this paper, we present our knowledge model for biomedical literature. We aim at delivering interoperable, interlinked, and self-describing documents in the biomedical domain. We applied our approach to the full-text, open-access subset of PubMed Central (PMC) [17]. PMC is a free full-text archive of biomedical literature; currently, it includes 1,679 journals. PMC provides an open-access subset; articles in this subset are still protected by copyright but are also available under the Creative Commons license, i.e., a more liberal redistribution is allowed. Articles are available as Extensible Markup Language (XML) files downloadable via File Transfer Protocol (FTP). In our approach, existing ontologies are brought together in order to facilitate the representation of sections in scientific literature as well as the identification of biologically meaningful fragments. These are pieces of text corresponding to proteins, chemicals, drugs, or diseases, among other biological concepts, within those previously identified sections. By delivering a semantic infrastructure for scientific publications, i.e., a semantic dataset, we are supporting interoperability as publications are linked to each other and to biological resources. By embedding biomedical literature in the WoD it is possible for users and developers to benefit from the advantages offered by the Linked Open Data (LOD) cloud.

## Results

**Figure 1:**
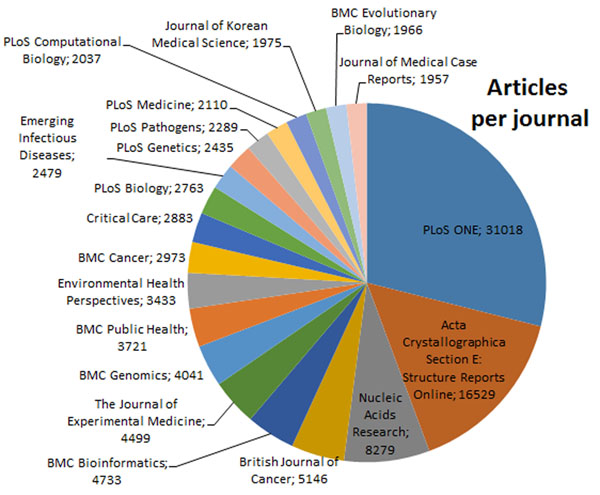
**Coverage per journal** Coverage per journal; only the top 20, corresponding to approximately 40% of the articles, are presented in this figure.

We are RDFizing biomedical literature and providing services in the form of an API. We define RDFize as a verb, meaning (i) to generate an RDF representation of something that was originally in a different format and (ii) to convert or transform to RDF. The Biotea project comprises and makes available (i) a set of RDF files generated from the open-access subset of PMC and enriched with semantic annotations, (ii) a Web Services API for querying the RDF dataset, (iii) a SPARQL Protocol and RDF Query Language (SPARQL) endpoint containing a subset of the RDF files as a proof of concept, (iv) an article-centric prototype that acts as an interface to the WoD, and (v) an implemented transformation process from our RDF files to Bio2RDF [18,19]. These services are available at http://biotea.idiginfo.org. Our dataset comprises 270,834 articles from PMC, distributed across 2,401 journals. About 40% of these articles correspond to 20 journals; those are presented in Figure 1.

Our RDFization process orchestrates ontologies such as the Documents Components Ontology (DoCO) [20], BIBO [3], DCMI [4,5], and FOAF [6,7]; these namespaces have been added to our SPARQL endpoint so that users do not need to define them as prefixes. Meaningful fragments within sections are automatically marked and enriched by adding annotations. Such annotations are structured with the Annotation Ontology (AO) [21]. In our model, we follow the four principles proposed by Tim Berners-Lee for publishing Linked Data [22]: (i) using Uniform Resource Identifiers (URIs) to identify things, (ii) using Hyper Text Transfer Protocol (HTTP) URIs to enable things to be referenced and looked up by software agents, (iii) representing things in RDF and providing a SPARQL endpoint, and (iv) providing links to external URIs in order to facilitate knowledge discovery.

The connectivity tissue supported by our dataset makes it possible to establish networks of associated concepts across papers (NACAP) [15]. In this way, the retrieved set can be represented as a graph where nodes are articles and shared terms are edges. This graph-based navigation allows users to realize how heavily two or more articles are interconnected as well as what terms are shared –see section “Gene-based search and retrieval, a first prototype” for a complete description of our current implementation. Our semantically enriched dataset also makes hierarchy-based searching possible. Based on the hierarchy of classes, retrieval can be widened to direct ascendants or narrowed to direct descendants; thus, the dataset can be navigated by going up or down in the hierarchy.

### RDFized PMC articles

**Figure 2:**
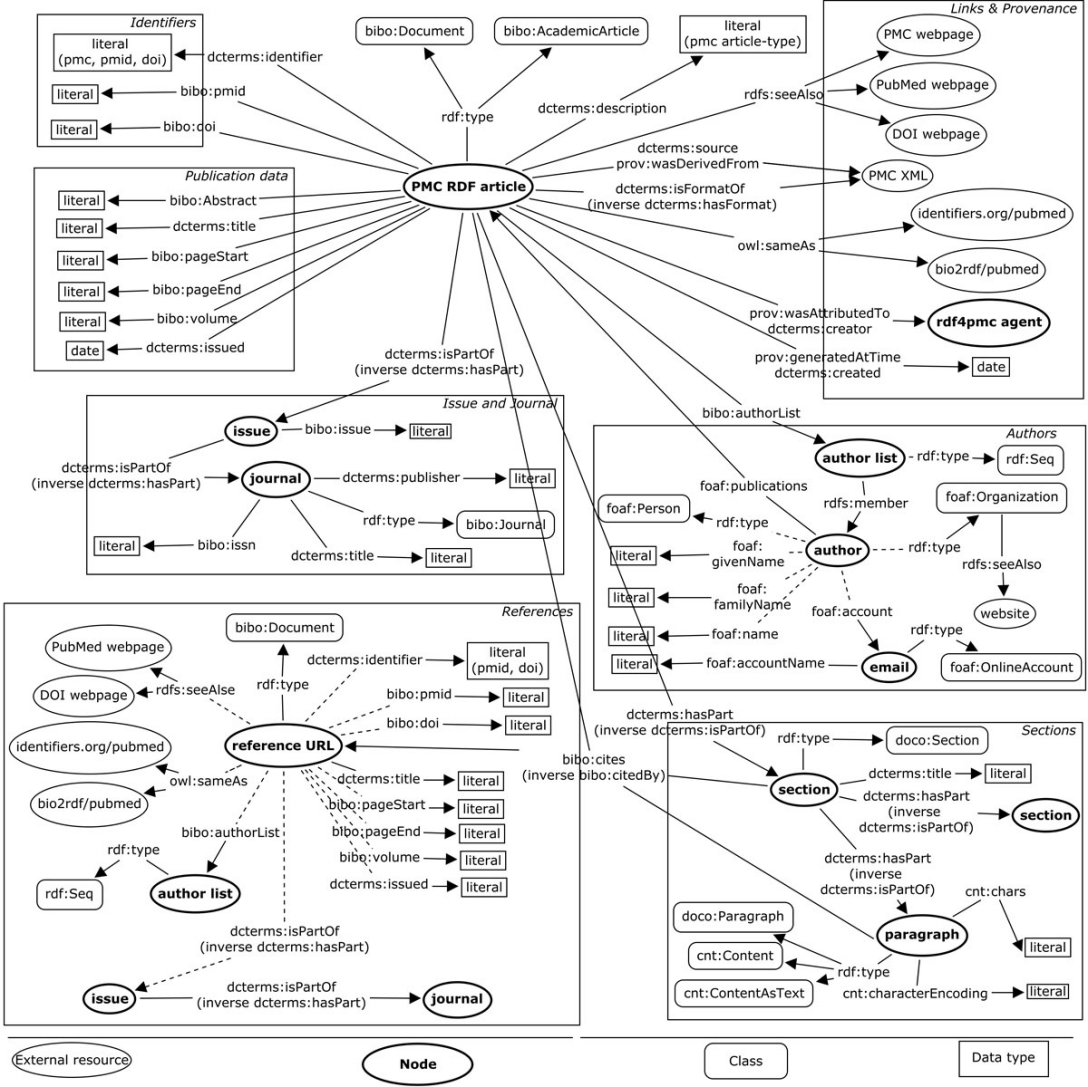
**RDF graph corresponding to an RDFized article** Graph for an RDFized article; the generated RDF includes identifiers, publication data, related links, authors, journal, and references. Provenance is also included. The order of the authors is not currently included in the RDFized version of the article.

We use BIBO and DCMI Terms to model the bibliographic metadata, DoCO to explicitly identify sections, and FOAF to identify authors and organizations. Figure 2 illustrates the graph that corresponds to bibliographic data, sections, and content; in this figure, we present the use of Digital Object Identifiers (DOIs), PubMed Ids, and PMC Ids as identifiers. Titles and keywords are represented by DCMI Terms. Relations to other resources representing the same entity are included as owl:sameAs; relations to webpages are included as rdfs:seeAlso. The abstracts are represented as BIBO elements and doco:Section. Published data is presented at the top of the figure; authors, which are found on the right side, are represented as a list of foaf:Person objects. Sections as well as references are also illustrated. Sections include a title and a set of paragraphs modeled as doco:Paragraphs and cnt:ContextAsText. The content *per se* corresponds to cnt:chars; the cnt namespace comes from the Representing Content vocabulary (CNT) [23]. References include metadata similar to that of the main article. It is possible to use this model to search articles with terms present in specific sections of the document. This is an improvement over traditional keyword-based search and retrieval tools, since such tools currently search for keywords either in the title, abstract, or entire text without the possibility of specifying a particular section. An example SPARQL query is provided in Table 1.

**Table 1:** Retrieving articles based on content

SPARQL query		Query expressed in natural language
	→	Retrieving PubMed identifier, article title, section title, and paragraphs for those articles containing the term “cancer” in any section whose title includes “introduction”

SPARQL queries can be used to retrieve metadata and content. It is possible to specify words and sentences that should appear in the text or in the section title.

### Semantically enriched content

**Figure 3:**
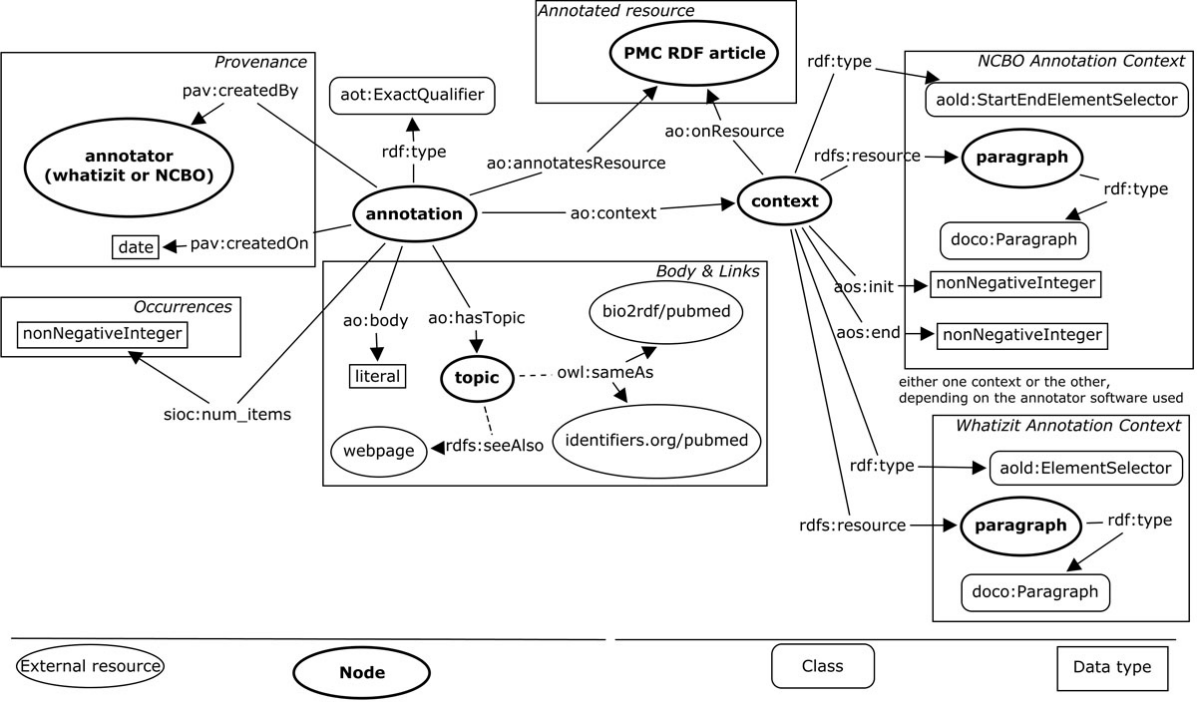
**RDF graph corresponding to annotations** The abstract and sections in the content are enriched with automatic annotations from either Whatizit or the NCBO annotator. For annotations generated by NCBO, it is possible to identify initial and final positions for the annotated term.

Figure 3 illustrates annotations generated by using text-mining tools, specifically Whatizit [24,25] and the NCBO Annotator [26]. The provenance information for annotations includes the creation date as well as the annotator used. Both the PMC article and the paragraphs where the term is located are identified. In addition, there is a set of ontologically related entities attached to the annotations. Furthermore, for terms that can be resolved by Bio2RDF [18,19] or identifiers.org [27], an owl:sameAs relation is used.

**Figure 4:**
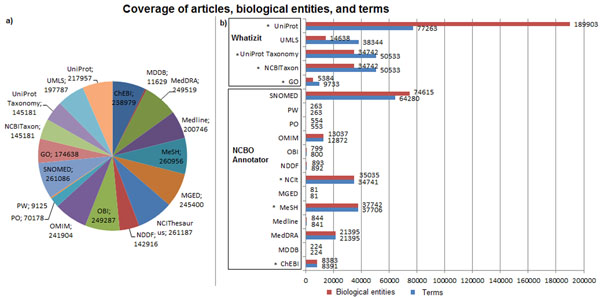
**Coverage per vocabulary** a) Number of articles covered per vocabulary. b) Number of biological entities and terms covered per vocabulary. Vocabularies that are part of Bio2RDF are indicated with a star (*); the text-mining tools are also identified.

Annotations in our dataset are distributed across eighteen different vocabularies. Thirteen of them were processed with the NCBO Annotator and five with Whatizit; seven of the vocabularies are also part of Bio2RDF. Figure 4 presents the coverage of articles, terms, and biological entities. In most of the cases, the number of terms identified in the articles is similar to the number of the entities associated with them. However, this is not true for UniProt, as Whatizit usually associates more than one protein per term; thus, the number of entities is about 2.5 times the number of terms. For instance, for PMC:1043860 "Scr" and "lacZ" were recognized by Whatizit as proteins; the first term was associated to two proteins –Q93CH6 and P09077, the second term was associated to seventeen proteins –Q59750, P30812, P23989, P06219, Q48727, Q56307, P70753, P26257, P81650, P0C1Y0, P77989, Q9K9C6, Q59140, O33815, P00722, Q47077, and Q1G9Z4. In the case of species, Whatizit recognizes scientific and common names; in this case, the number of identified terms was higher. For diseases, Whatizit identifies names and abbreviations from the Unified Medical Language System (UMLS), also resulting in more terms than entities.

**Figure 5:**
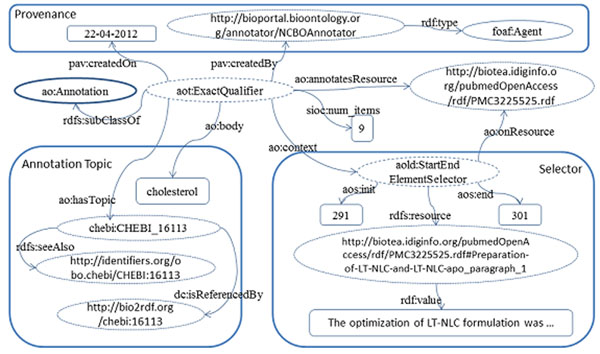
**Annotation for a chemical entity** Annotation for a chemical entity: *cholesterol*. The term is located in the first paragraph of the section titled “Preparation of LT-NLC and LT-NLC-apo”, from position 291 to position 301; the corresponding CHEBI term is CHEBI_16113.

In Figure 5, we present an example RDF representation of an annotation from the article PMC:3225525, “*Preparation and Characterization of a Lovastatin-Loaded Protein-Free Nanostructured Lipid Carrier Resembling High-Density Lipoprotein and Evaluation of its Targeting to Foam Cells*”. A fragment of the introduction, “*cholesterol*”, has been annotated with the ontological term chebi:CHEBI_16113. ChEBI terms are identified by means of the NCBO annotator, represented as a foaf:Agent in the provenance section. The NCBO annotator also provides the start and end positions within the text. Additional information about the topic is provided by the rdfs:seeAlso relation. A query that retrieves documents with a specific chemical term is demonstrated in Table 2.

**Table 2:** Retrieving articles based on annotations

SPARQL query		Query expressed in natural language
	→	Retrieving PubMed identifier for those articles that have been semantically annotated with the biological entity CHEBI:60004. The semantic annotation comes from the occurrence of the term “mixture” in any paragraph of the retrieved articles.

As content has been semantically enriched, it is possible to retrieve articles based on either the annotated terms, e.g., “mixture,” or their corresponding biological entities, e.g., CHEBI:60004.

### Biotea API

In addition to downloadable RDF documents, we also provide a web services API for the Biotea collection. The API is available at http://biotea.idiginfo.org/api and provides services for information retrieval by using predefined indexes encapsulated behind a set of simple read-only Representation State Transfer (REST) services. The REST mechanisms facilitate retrieval of documents through the discovery of terms, topics, and vocabularies within the documents. The following specific services are available:

• http://biotea.idiginfo.org/api/→Provides information about the API;

• http://biotea.idiginfo.org/api/topics→ Query collection based on topics;

• http://biotea.idiginfo.org/api/terms→ Query collection based on terms;

• http://biotea.idiginfo.org/api/vocabularies→Query collection based on vocabularies; and

• http://biotea.idiginfo.org/api/documents→Retrieves RDFized documents.

**Figure 6:**
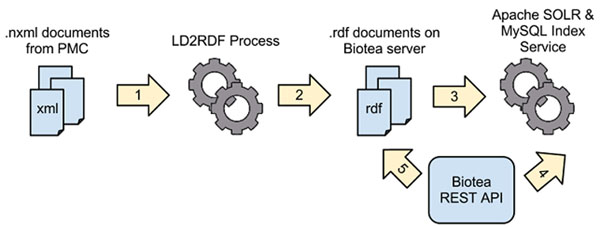
**Biotea API, from RDF to indexes and web services** The RDF4PMC process takes in NXML files provided by PMC as input (1) and generates RDF files (2). Apache SOLR and MySQL are used to index the RDF files, which occurs as a separate, asynchronous process (3). Users and other applications can then use the Biotea REST API to retrieve data from the RDF files (4 and 5).

These services can be used to retrieve information from the RDF graph in a number of ways. A full list of services and their parameters is available at http://biotea.idiginfo.org/api Cases currently supported by the API are summarized in Table 3. The API uses several open source technologies, including Apache SOLR, Apache HTTPD, MySQL, and the PHP Silex framework. Flat-file RDF documents generated by the RDFization process are asynchronously indexed by SOLR & MySQL and made available via the PHP Silex application. See Figure 6 for an illustration of the indexation process.

**Table 3:** API retrieval support

Retrieval	Service
A list of terms and their related topics	http://biotea.idiginfo.org/api/terms

A list of topics and their related vocabularies	http://biotea.idiginfo.org/api/topics

All topics related to a term	e.g., http://biotea.idiginfo.org/api/topics?term=cancer

All vocabularies related to a term	e.g., http://biotea.idiginfo.org/api/vocabularies?term=cancer

All terms that start with a specific string (for autocompletion)	e.g.,http://biotea.idiginfo.org/api/terms?prefix=canc

All topics related to a vocabulary	e.g., http://biotea.idiginfo.org/api/topics?vocabulary=po

RDF of articles that include a term	e.g., http://biotea.idiginfo.org/api/articles?term=cancer

Count of RDF of articles that include a term	e.g., http://biotea.idiginfo.org/api/articles?term=cancer&count=true

A list of vocabularies and their prefixes	http://biotea.idiginfo.org/api/vocabularies

RDF of articles that include a vocabulary	e.g., http://biotea.idiginfo.org/api/articles?vocabulary=po

### Gene-based search and retrieval, a first prototype

Developers can make use of our API and downloadable dataset to create web applications and mash-ups. In order to illustrate some of the possibilities, we have developed a prototype application that makes it possible for users to search for human genes in PMC. Unlike search & retrieval tools that return only the target documents from a given search, our prototype search tool returns an enriched visualization of the annotated terms and the biological entities related to them. Biomedical entities are fully identified, enabling the association of specific tools to enhance the reading experience, e.g., sequence browsers, 3D viewers, etc. Entities are also linked to resources such as Bio2RDF, thus immersing the content in the WoD.

**Figure 7:**
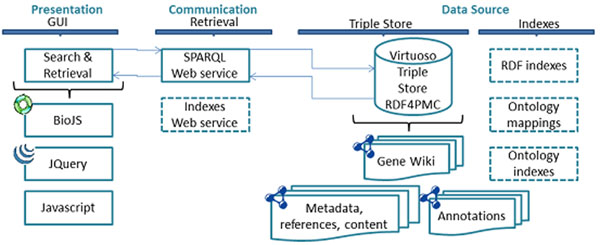
**Prototype architecture** The flow of data is indicated by the arrows between layers. Current components are shown in continuous-lines; future components are in dashed-lines.

The architecture for this prototype consists of two layers, Presentation and Communication, as presented in Figure 7. The Presentation layer is a web-based Search & Retrieval interface. It incorporates JavaScript technologies such as JQuery [28] and BioJS [29]. The Presentation layer manages the organization of the data; it relies on the Communication layer in order to retrieve the required information from our SPARQL endpoint, which also hosts the GeneWiki [30] dataset. The performance of retrieval could potentially be improved by using indexes over the RDF, which are available via our Web Services API. Additional ontology mappings and indexes would also facilitate more advanced search options.

Users initiate a search by typing the name of a gene. From the gene name, the corresponding protein accession is retrieved from the GeneWiki RDF. GeneWiki is a Wikipedia-based project comprising over 10,000 pages about human genes, and it includes mappings to proteins and diseases. Protein accessions in GeneWiki follow the Universal Protein Resource (UniProt) nomenclature. UniProt is a consortium that provides free access to a high-quality collection of protein sequences and functional annotations [31].

**Figure 8:**
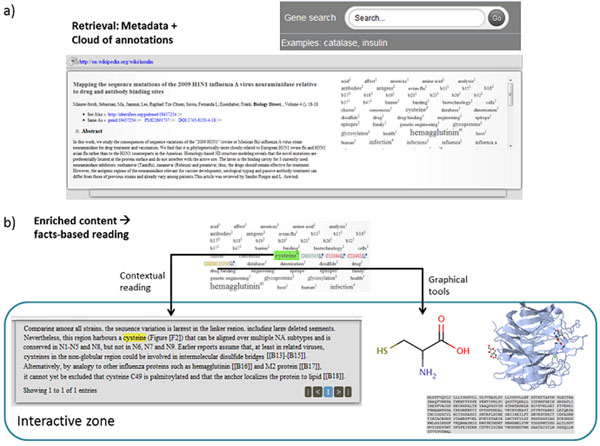
**Our prototype at a glance** a) Search and retrieval based on human gene names; the term is initially resolved against GeneWiki, the associated UniProt accession is then used in the query. The resulting set includes publication metadata, abstract, and a cloud of annotations. b) Enriched content based on annotations is displayed in the interactive zone; this may be the annotated paragraph, a chemical entity, or protein related information.

An example is presented in Figure 8a. In this example, all available articles annotated with “*Insulin*” are retrieved and presented in alphabetical order. Initially, links to GeneWiki, identifiers.org, PubMed, PMC, and DOI are displayed in the interface, along with title, authors, and abstract A tag cloud is also displayed; it weighs each term based on the number of biological entities associated with it. Whenever a term in the cloud is selected, the vocabularies used to define this term are displayed. Once a vocabulary has been selected, the biological entities for that vocabulary are also displayed. The interactive zone found in Figure 8b changes depending on the selection in the tag cloud: (i) when a term is selected, all paragraphs containing the term are displayed; a simple navigation bar allows users to move from one paragraph to the other. Similarly, (ii) when a biological entity is selected, relevant information is displayed, e.g., sequences and 3D structures for proteins, structures for chemicals, or images for species.

Unlike tools such as Reflect [32], our prototype makes use of BioJS components that are able to interact with each other. For instance, whenever the selection over a protein sequence changes, the interface highlights the corresponding amino acids in the 3D structure. As data types are fully identified, further manipulation becomes possible. In this way, we deliver a rich and interactive user experience.

**Figure 9:**
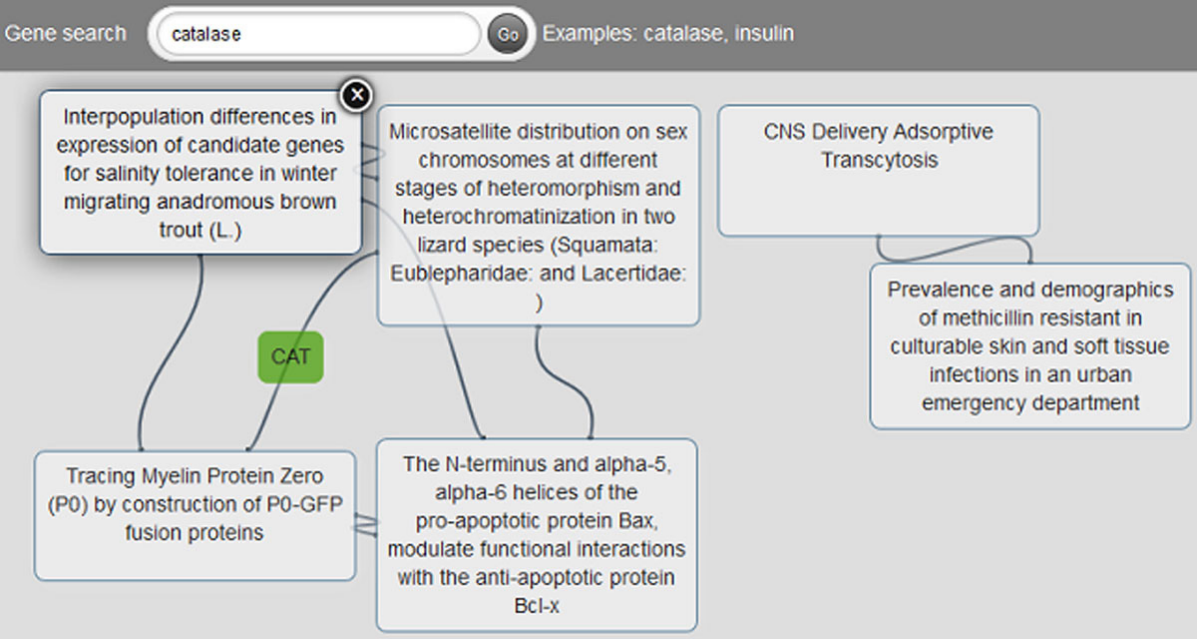
**NACAP in action, graph-based retrieval** Graph-based retrieval for the terms “*catalase*”; only shared terms with more than 30 associated biological terms are included in the results.

Graph-based navigation of the data is also possible; see Figure 9 for an illustration. As in the previous use-case, when a user searches for a human gene, for instance “*catalase”*, instead of a table, the result is now displayed as a graph with articles as nodes and terms as edges. In order to facilitate the identification of the articles, the title is displayed. Users can easily remove articles from the results, so they can focus on those that are more interesting for them. As presenting all the shared terms can be overwhelming, we display only terms with more than 30 associated biological entities. The actual term represented by an edge is displayed on mouse-over. Some of the improvements we are working on for the graph-based view include: (i) defining whether terms or vocabularies should be used as edges, (ii) establishing the minimum and maximum weight of the edges to be displayed –currently the weight is determined by the number of biological entities associated with a term and is fixed to 30, (iii) displaying tool tips with associated biological entities, i.e., a cloud of tags, on mouse-over, (iv) filtering of specific vocabularies so the user can focus on those in which s/he is more interested, (v) paginating the results so not all the articles are displayed at the same time, and (vi) adding the interactive zone for a user-selected article so users will have the same functionality as they currently have in the linear results.

### Integration with Bio2RDF

Bio2RDF [18,19] is a project that makes biomedical data available by using Semantic Web technologies such as RDF and SPARQL. Bio2RDF brings together information from diverse public databases such as Kyoto Encyclopedia of Genes and Genomes (Kegg), Protein Databank (PDB), UniProt, NCIt, and PubMed, amongst others. Both Bio2RDF and RDF4PMC aim to support biological knowledge discovery, the former by providing a single access point to several biomedical data sources, and the latter by delivering a semantically enriched information layer on top of PMC articles. Our RDFized PMC articles are semantically rich and deeply related to biomedical data sources available via Bio2RDF, and the WoD at large, so ensuring that they are fully compliant with Bio2RDF is critical for bridging the gap between research articles and biomedical data sources. Mappings and processes will be available via the Bio2RDF GitHub repository.

## Discussion

We have generated a semantically enriched version of PMC. Our model makes extensive reuse of existing vocabularies. Annotations are scaffolded by using the AO, domain knowledge is identified by means of domain ontologies, and documents are structured by using DOCO, BIBO, DCMI Terms, and others. Some of the difficulties we have had are: (i) at least four different formats are used to model references in PMC XMLs, (ii) authors’ names are represented with initials and last name, making it difficult to disambiguate them, (iii) FOAF for authors and institutions are not provided, and (iv) annotation services were sometimes unavailable during processing –services are not always reliable. In order to deal with different reference styles, we create specific methods for each one and transform them into a common RDF model following the recommendations from BIBO. We also create FOAF elements for authors and institutions and assign them resolvable URIs; in this way, it would be possible to use tools such as http://sameas.org[33] for defining relationships between our FOAF and the WoD. When combined with social mechanisms, this technique may be used to disambiguate article contributors; authors could claim publications, so the FOAF could be consolidated. The problems with the annotation services were resolved by reprocessing files whenever needed. For the Gene Ontology (GO), National Drug File – Reference Terminology (NDFRT), Foundational Model of Anatomy (FMA), Symptom Ontology (SYMP), and the International Classification of Diseases, vr.10 (ICD10), it was necessary to update some of the term-related URIs and reprocess the annotations. GO was reprocessed with Whatizit; we are using the NCBO annotator for FMA, NDFRT, SYMP, and ICD10.

We have generated an interoperable semantic dataset. Models such as that of NPG do not link to existing vocabularies, e.g., MeSH, in a semantic way. Instead, they include plain literals, making it difficult to use this information for knowledge discovery. Our model links to well-known vocabularies relevant in the biomedical domain. Similar to the NPG experience, we also rely on ontologies such as BIBO in order to model metadata. Since we are targeting only open-access documents within PMC, we also include the content of the document. Similar to Reflect and UTOPIA, we integrate content from different resources into scientific publications; in our dataset, this is achieved by means of the semantics annotations added to the articles. In our case, this integration is persistent and interoperable with other resources.

Using ontologies to annotate concepts in scientific publications is a common practice in PubMed; curators use MeSH to annotate PubMed. Finding hidden relations by using semantic annotations has been reported by several authors in the biomedical domain [34-36]. For instance, patterns across the MeSH terms have been used to identify potential new associations between drugs and diseases [36]. Also, annotations shared by a group of genes have contributed to identify possible relationships between these genes [34,35]. The entity recognition systems we are using, namely Whatizit and the NCBO Annotator, deliver annotations as HTTP URIs; in this way, annotated concepts can be referenced and searched by software. By structuring annotations in RDF, we are facilitating interoperability between the content and the WoD. For instance, it is possible to retrieve documents, focusing on the section “*Materials and Methods”*, about gene expression in whole blood from cancer patients and complementing the results with information about drugs and chemical entities that might act on a certain protein. The possibilities are limited to existing links in the LOD cloud and the availability of such resources over SPARQL endpoints.

Scientific literature is intrinsically related to each other via citations. It has been considered that two citing articles are similar to the extent that they cite the same literature [37]; such is the nature of citation-based approaches (e.g., co-citation analysis, bibliographic coupling). Text-based approaches (e.g., term frequency-inverse document frequency (tf-idf), latent semantic analysis) can also be used to measure similarity across documents [38]. Lewis [39] suggests text similarity as an alternative search mechanism for the Medline database [40]; articles are initially grouped by means of a keyword-based algorithm and then ordered following a similar algorithm based on sentence alignment. McSyBi [41] utilizes clustering techniques in order to make sub-topics explicit from a set of citation data retrieved from PubMed; clusters are created based on information from the title and the abstracts. Users can modify the cluster by specifying a MeSH term or a UMLS Semantic Type; this makes it easier for users to obtain different graphs that can be analyzed from different perspectives depending on the terms used as secondary input. Unlike McSyBi, PuRed-MCL [42] does not analyze the content of the article; instead, it relies on a set of pre-computed relations from PubMed. Those related documents are then used to build a graph that is processed by a clustering technique. Clusters and documents are annotated with MeSH and chemical substances; visualization is also supported. Most of the investigated approaches address the problem by using relatively small annotation graphs with few ontologies. As our dataset comprises annotations from over 20 ontologies, it provides an ideal playground for semantic similarity analysis across biomedical literature.

**Figure 10:**
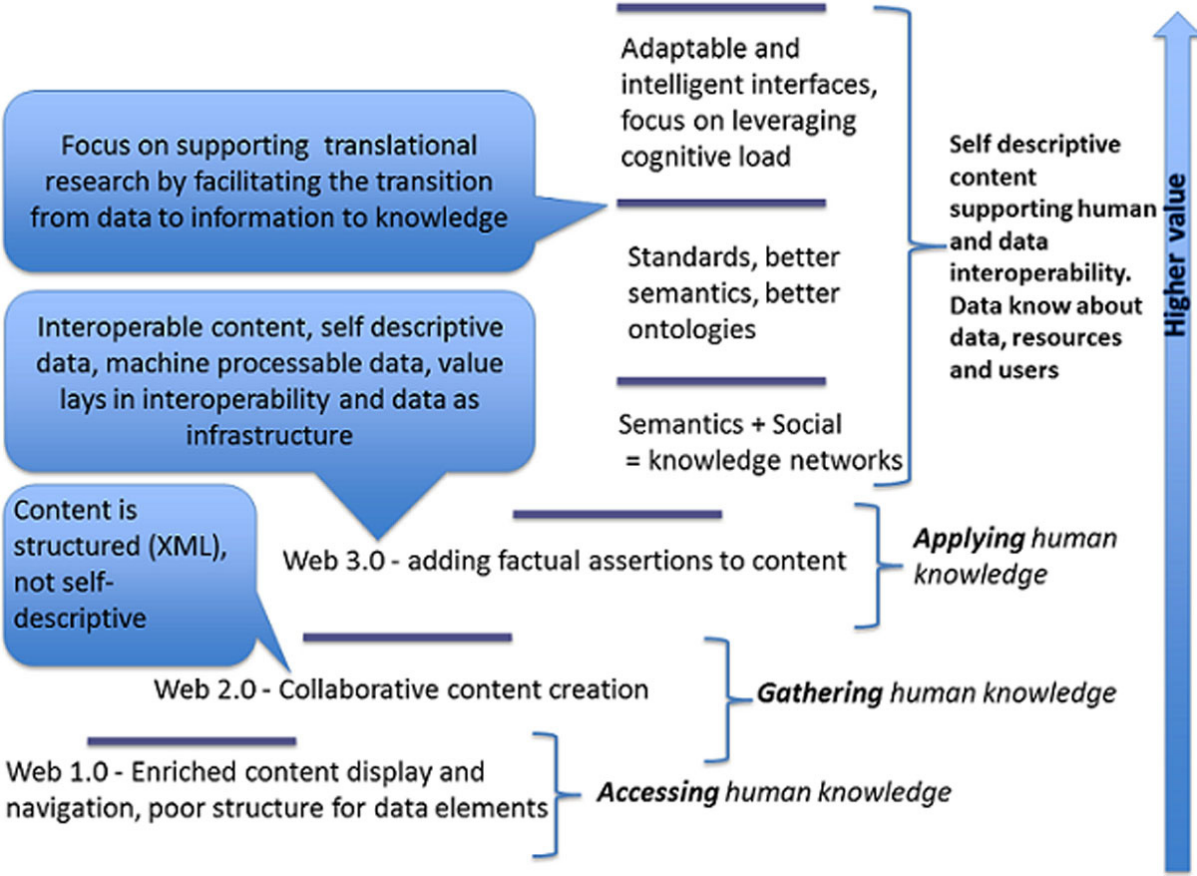
**From Web 1.0 to Web 3.0 and beyond** An evolutionary glimpse.

Our model is flexible; adding a new annotator, e.g., an additional text-mining tool, is possible. It is also feasible to use other input sources, e.g., XML or other formats from different publishers. Our model delivers a semantically rich and highly linked dataset; value is added because the dataset is fully immersed in the WoD. Our dataset delivers self-describing documents, making it possible for new knowledge to be discovered as a consequence of the enrichment of metadata and the interrelated nature of the semantic model. Our approach is a natural consequence of the evolution of the Web; Figure 10 illustrates how data gains value as it is better described and therefore more interoperable. Data linked to other data makes for more useful and universally applicable data. Such is, we argue, the value behind our semantically enriched PMC.

## Conclusions

Our methods as well as the resulting dataset are an important part of the semantic infrastructure for PMC. We provide (i) the transformation into RDF from the original PMC files, (ii) the annotation of the RDF, and (iii) an API which makes that data available. New vocabularies as well as annotators can easily be plugged in, making it possible to enrich the semantics of the dataset by supporting use-cases not covered by those vocabularies and annotators that were initially used. Our approach is useful for both open and non-open access datasets; since the content is clearly identified and enriched with specialized vocabularies, publishers may decide what to expose via RDF and what content to make available.

To ensure the reproducibility of science, we envision that scientific articles will provide access to raw data and to computer-understandable descriptions of methodologies in order to support the recreation of the experiments being described. To aid in resolving inconsistencies, we expect in the future to relate and compare information across multiple documents. Semantic Web technologies should help to deliver a self-descriptive document that makes it possible to improve the user experience and change our understanding of scholarly communication. There should be a community-based platform providing FOAF for authors and institutions; such a platform could easily be part of publication submission systems. In this way, disambiguating authors will become much simpler.

## Methods

### Materials: Ontologies and text-mining tools

We use BIBO, DCMI Terms, and the Provenance Ontology (PROV-O) [43] to model the bibliographic metadata. BIBO [3] provides classes and properties to represent citations and bibliographic references. BIBO can be used to model documents and citations in RDF or to classify documents within a hierarchy. BIBO reuses concepts from DCMI and PRISM –an XML specification that defines a controlled vocabulary for managing, aggregating, and publishing content; PRISM is also used in the RDF dataset provided by NPG. Dublin Core (DC) [4,5] offers a domain-independent vocabulary to represent metadata; such vocabulary aims to facilitate cross-resource exploration. The DC vocabulary was initially released in 1995, and in 2008 the DCMI was created. It aims to provide an interoperable-online set of metadata standards. PROV-O is a working draft from the World Wide Web Consortium (W3C); W3C is the main international organization for standards in the World Wide Web (WWW). PROV-O provides classes and properties to represent and interchange provenance data.

Some of the properties provided by BIBO are similar to those found in the DCMI Terms; BIBO inherits some properties from DCMI Terms. We use bibo:pmid and bibo:doi as publication, domain-specific identifiers. We have also included dcterms:identifier because, being a domain-independent property, it is widely used and facilitates compatibility with existing RDF datasets such as that from NPG. Some of the properties provided by PROV-O are similar to those available in DCMI Terms. For instance, prov:wasAttributedTo is similar to dcterms:creator, and prov:generatedAtTime is similar to dcterms:created. We have included both PROV-O and DCMI Terms properties. Similar to the previous case, PROV-O is more specific, whereas DCMI Terms is more used.

In BIBO, authors are modeled either as rdf:List or rdf:Seq. In both cases, the range should be rdfs:Resource [44]. By doing so, authors have been modeled as resources rather than as plain text. We use FOAF [7] to identify the affiliations of authors. Specifically, we use foaf:Person and foaf:Organization. FOAF provides a set of classes and properties to represent people and their connections to other people, organizations, and resources, e.g., publications. FOAF integrates information related to social networks. Such networking is also identifiable in publications; authors collaborate with co-authors and are affiliated to organizations. Authors could be represented as dcterms:Agent. However, we have used FOAF because it is more detailed and explicit. It includes elements such as first and last name, the institution to which the author belongs, personal homepage, and email account.

We use DoCO [20] to explicitly identify sections and paragraphs. CNT [23] is being used to represent the actual content in the paragraphs. DoCO provides a structured vocabulary written in OWL 2 DL of document components, both structural (e.g. block, inline, paragraph, section, chapter) and rhetorical (e.g. introduction, discussion, acknowledgements, reference list, figure, appendix). The content of paragraphs is modeled with CNT. CNT is a working draft from W3C – last released in May 2011, it aims to provide a flexible vocabulary for the representation of any type of content, e.g., text, XML, images, etc. It includes the encoding character, making it easier for machines to process the content.

In order to identify biological terms, we use two text-mining tools: Whatizit [24,25] and the NCBO Annotator [26]. Both tools are based on exact string matching and pre-defined dictionaries. Whatizit is based on monq.jfa [45], an open source Java library that binds regular expressions to actions; these actions are automatically executed whenever there is an exact string match between the dictionary and the processed text. In the case of Whatizit, an XML tag is added around the match. By doing so, relevant biological identifiers such as UniProt accessions and ChEBI and GO identifiers are added. The NCBO Annotator is based on Mgrep [46]. Similar to Whatizit, the NCBO annotator identifies terms and associates them with biological entities. However, the NCBO Annotator also utilizes to its advantage the hierarchy in the vocabularies used for the association. It adds siblings and maps to equivalent terms in other ontologies.

The identified terms are represented in RDF following the model proposed by the AO [21]. The AO facilitates the representation of annotations on static resources. The AO supports annotations expressed as plain text as well as those coming from ontologies. Annotations can be attached to a whole resource but also to portions of it, e.g., sentences, paragraphs, sections, etc. Annotations on specific parts of a document make use of selectors; a selector identifies a portion within the text. Depending on its nature, it may be aos:TextSelector to identify an exact match or aos:StartEndSelector for the initial and final position in the annotation. AO uses FOAF for annotators.

### PMC RDFization process

**Figure 11:**
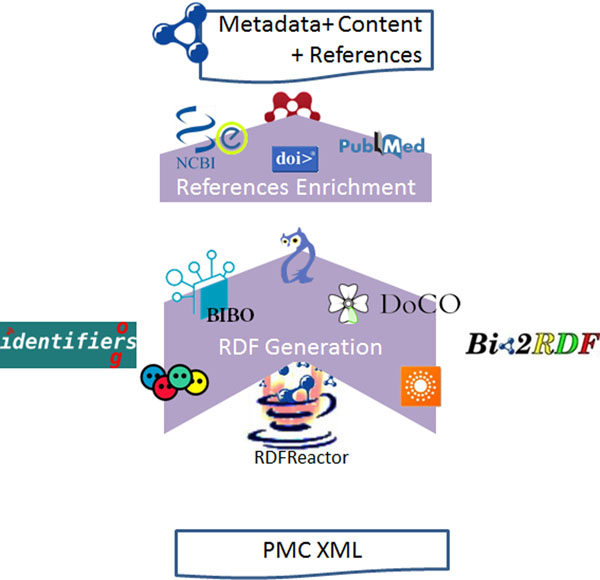
**RDFization process** The XML from PMC is transformed to RDF; RDFReactor is used to automatically generate java classes for the ontologies. XML elements are transformed into RDF, and, whenever possible, incomplete references are enriched. Common ontologies used in bibliographic data such as FOAF, DCMI Terms, BIBO, and DoCO are used.

The workflow that we followed to generate the RDF files for PMC articles is depicted in Figure 11. The main input for our process is the XML offered by PMC for open- access articles. We are also using available vocabularies to represent the metadata and content in RDF; such vocabularies have been mapped to Java classes by using RDFReactor [47]. The article itself is modeled as bibo:Document; whenever it is possible, a more accurate class is also added, e.g., bibo:AcademicArticle for research articles. Publisher metadata is modeled using BIBO, including publisher name, the International Standard Serial Number (ISSN), volume, issue, and starting and ending pages. Authors are modeled as a bibo:authorList, where each member is a foaf:Person. Abstract and sections are modeled as a doco:Section with a cnt:chars containing the actual text with formatting omitted. Well-known identifiers such as PubMed and DOI are included in the output; thus, it is possible to track the original source of the article. The same principle is also applied to the references. For incomplete references, e.g., “Allen, F. H. (2002). ActaCryst. B58, 380-388” in PMC:2971765, it is possible to use services such as Mendeley [48], CrossRef [49], and eFetch [50] in order to complete the information so title and identifiers can be added. The references are modeled as bibo:Document; the relations used are bibo:cites and bibo:citedBy. References are available for both the document and the section level. We produce one file per publication; for example, for http://www.ncbi.nlm.nih.gov/pmc/articles/PMC2971111 the http://biotea.idiginfo.org/pubmedOpenAccess/rdf/PMC2971111 is generated.

### PMC Annotation process

**Figure 12:**
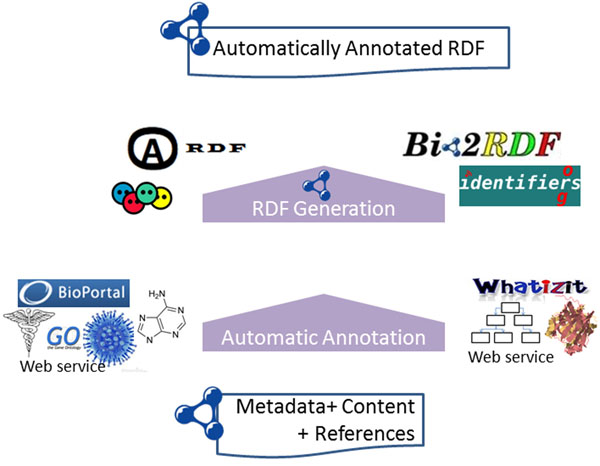
**Orchestrating ontologies and annotation services** The abstract and content from the paper, in RDF format, are annotated with Whatizit and the NCBO annotator. The former is used for proteins and species; the latter is used for drugs, genes, chemicals, and diseases. Annotations are modeled with AO. Links to identifiers.org and Bio2RDF are also included.

Once the RDF has been generated, the abstract and sections are enriched with automatic annotations modeled with AO. These automatic annotations are generated by the NCBO Annotator [26] and Whatizit [24,25]; see Figure 12 for more information. Adding the annotations to the RDF rather than to the original XML makes it easier when applying the same process to compatible RDFs from other publishers. For each doco:Section element in the RDF representation of an article, our process applies both the NCBO Annotator and Whatizit to produce two RDF files, one for each annotator. Different pipelines are possible in Whatizit; for our dataset, we have used *UkPmcAll* since it is recommended when dealing with proteins and genes.

The NCBO Annotator is used with the following ontologies:

• ChEBI for chemicals;

• Pathway, and Functional Genomics Data Society (MGED) for genes and proteins;

• Master Drug Data Base (MDDB), NDDF, and NDFRT for drugs;

• SNOMED, SYMP, MedDRA, MeSH, MedlinePlus Health Topics (MedlinePlus), Online Mendelian Inheritance in Man (OMIM), FMA, ICD10, and Ontology for Biomedical Investigations (OBI) for diseases and medical terms;

• PO for plants; and

• MeSH, SNOMED, and NCIt for general terms.

Whatizit is used for GO, UniProt proteins, UniProt Taxonomy, and diseases mapped to the UMLS; UniProt taxa are also mapped to NCBI Taxon vocabulary. ChEBI, GO, and organisms are supported by both NCBO and Whatizit. For ChEBI, we chose the NCBO Annotator because it is faster than Whatizit. For GO, we chose Whatizit because it allows the use of either the Foundry-compliant URIs or the OBO legacy ones [51]. In order to better align our effort with Bio2RDF, we are using the OBO legacy URIs. For organisms, we chose Whatizit because it recognizes more organisms than the NCBO Annotator; for example, neither “human” nor “mouse” was recognized by NCBO in any of our tests. Additionally, we included links to Bio2RDF for ChEBI, GO, MeSH, NCIt, UniProt proteins, UniProt Taxonomy, and NCBI Taxon.

In order to specify the location of an annotated term in a document, we have extended AO. Our extension makes it possible to select portions of text and represent them as RDF literals; the property whose object is the literal must be used only once in the annotated element. For example, the literal cnt:chars can only occur once per each doco:Paragraph in the article (<a doco:Paragraph> cnt:chars <a literal>). Extensions are shown below:

• aold:ElementSelector → identifies an exact text in a literal, e.g.,cnt:chars, in an RDF element (extends aos:TextSelector); and

• aold:StartEndElementSelector → like the previous one but also includes the start and end positions of the snippet in the text (extends aos:StartEndSelector).

### Bio2RDF mapping

**Figure 13:**
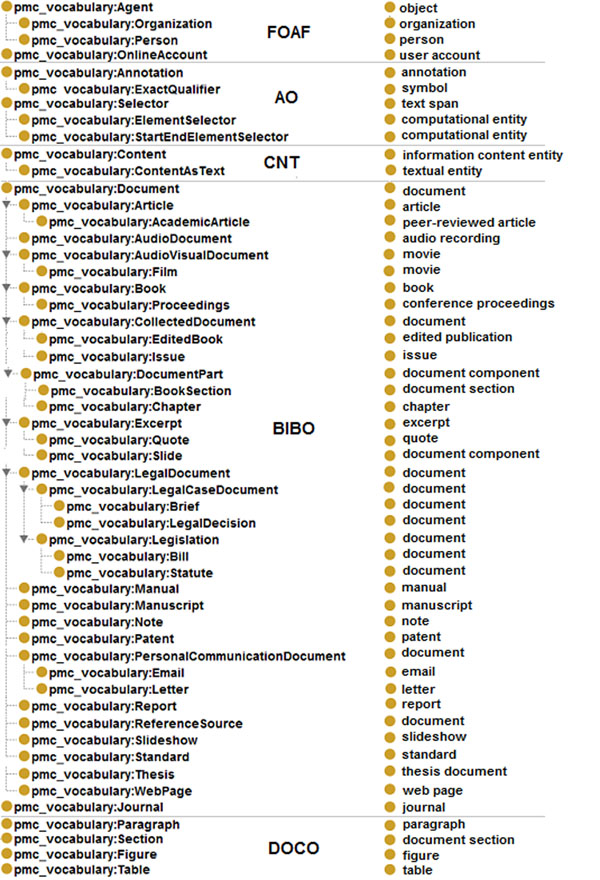
**Classes mapping to Bio2RDF** Classes from BIBO, DoCO, FOAF, and other vocabularies used during generation of the RDF have been mapped to SIO –the ontology currently used by Bio2RDF.

**Figure 14:**
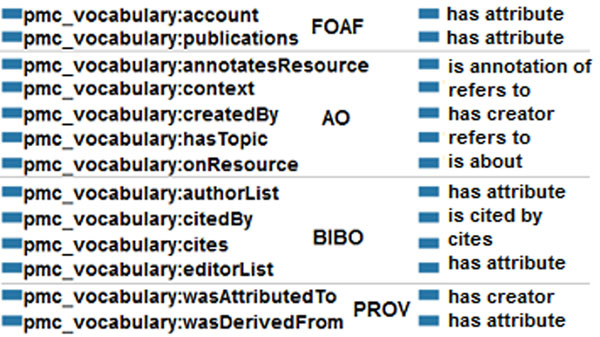
**Properties mapped to Bio2RDF** Mappings for object properties; data type properties are not included as all of them were mapped to the ‘has value’ property in SIO.

In order to integrate RDF4PMC with Bio2RDF, we have mapped the vocabularies used in our dataset to the Semantic Science Integrated Ontology (SIO) vocabulary. Consequently, the vocabularies use a structure compatible to datasets available in Bio2RDF. We have initially created an ontology, named *pmc_vocabulary*, with all the classes and terms used in our dataset. This ontology was then mapped to SIO; figure 13 lists the mapping for the classes. Object properties and data type properties have also been mapped; see Figure 14. Object properties mainly extend sio:000008 –“has attribute”; however, for some properties referencing other resources, we are using sio:000212 –“is referenced by”, and sio:000628 –“refers to”. All data type properties extend from sio:000300 –“has value”. For simplicity, original domains and ranges have not been mapped. Based on the defined mappings, the RDF files in our dataset are transformed into a format compliant with Bio2RDF. This transformation is mainly done by using the “construct” command offered by SPARQL.

## List of Abbreviations

AO: Annotation Ontology; API: Application Programming Interface; BIBO: Bibliographic Ontology; ChEBI: Chemical Entities of Biological Interest; CNT: Representing Content in RDF 1.0; DC: Dublin Core; DCMI: Dublin Core Metadata Initiative; DoCO: DoCO, the Document Components Ontology; DOI: Digital Object Identifier; FMA: Foundational Model of Anatomy; FOAF: Friend of a Friend Ontology; GO: Gene Ontology; GWT: Google Web Toolkit; HTTP: Hyper Text Transfer Protocol; ICD10: International Classification of Diseases - vr.10; ISSN: International Standard Serial Number; Kegg: Kyoto Encyclopedia of Genes and Genomes; LD: Living Document Project; LOD: Linked Open Data; MDDB: Master Drug Data Base; MedDRA: Medical Dictionary for Regulatory Activities; MedlinePlus: MedlinePlus Health Topics; MeSH: Medical Subject Headings; MGED: Functional Genomics Data Society; NACAP: Networks of Associated Concepts Across Papers; NCBI: National Center of Biotechnology Information; NCBO: National Center for Biomedical Ontology; NCIt: National Cancer Institute Thesaurus; NDDF: National Drug Data File; NDFRT: National Drug File - Reference Terminology Source; NPG: Nature Publishing Group; OBI: Ontology for Biomedical Investigations; OMIM: Online Mendelian Inheritance in Man; PDB: Protein Data Bank; PDF: Portable Document Format; PMC: PubMed Central; PRISM: Publishing Requirements for Industry Standard Metadata; PROV-O: Provenance Ontology; REST: Representation State Transfer; RDF: Resource Description Framework ; SDL: Semantic Digital Libraries; SIO: Semantic Science Integrated Ontology; SNOMED: Systematized Nomenclature of Medicine; SPARQL: SPARQL Protocol and RDF Query Language; SYMP: Symptom Ontology, tf-idf: Term frequency-inverse document frequency; UMLS: Unified Medical Language System; UniProt: Universal Protein Resource; URI: Uniform Resource Identifier; WoD: Web of Data; XML: Extended Markup Language.

## Competing interests

We declare having no competing interests.

## Authors' contributions

LJGC was the main developer of the RDFization process for PMC; she conceptualized the models supporting this work and the mappings to Bio2RDF. CML developed the indexing process as well as the related web services; he also collaborated in the final proofreading. AG conceived the original idea, contributed to the models and mappings, participated in the software development process, and supervised this project; he retained the overall responsibility of the final edition. AG and LJGC are the main contributors to this work.

## Declarations

AGC, CM and the the journal submission have been funded by US DoD Grant MOMRP Grant w81xwh-10-2-0181. LJG has been self-funded. During the early stages of this project, 2010-2011 AGC was self-funded.

This article has been published as part of *Journal of Biomedical Semantics* Volume 4 Supplement 1, 2013: Proceedings of the Bio-Ontologies Special Interest Group 2012. The full contents of the supplement are available online at http://www.jbiomedsem.com/supplements/4/S1
